# Chinese medicine therapies for neurogenic bladder after spinal cord injury: A protocol for systematic review and network meta-analysis

**DOI:** 10.1097/MD.0000000000027215

**Published:** 2021-09-17

**Authors:** Zhihong Zhu, Yue Zhuo, Haitao Jin, Boyu Wu, Zhijie Li

**Affiliations:** a Clinical College of Traditional Chinese Medicine, Hubei University of Chinese Medicine, Wuhan, Hubei, China; b College of Acupuncture-Moxibustion and Tuina, Hunan University of Chinese Medicine, Changsha, Hunan, China; c Department of Neurology, Wuhan Hospital of Traditional Chinese Medicine, Wuhan, Hubei, China.

**Keywords:** network meta-analysis, neurogenic bladder, protocol, spinal cord injuries, systematic review, traditional Chinese medicine

## Abstract

**Background:**

Neurogenic bladder (NB), a refractory disease, is characterized by voiding dysfunction of bladder and/or urethra, and spinal cord injury (SCI) is a common cause. Chinese medicine therapies have been applied extensively in the treatment of NB, especially in China, and the results are promising but varying. Thus, the aim of this work is to assess the efficacy and safety of various Chinese medicine therapies for NB after SCI.

**Methods:**

A retrieval will be performed in 8 online databases (the Cochrane Library, Web of Science, PubMed, EMBASE Database, China Biological Medicine Database, Chinese Scientific Journals Database, Wan Fang databases, and China National Knowledge Infrastructure) from their inception throughout June 2021. Only randomized controlled trials of testing Chinese medicine therapies for NB after SCI will be enrolled. The outcome indicators measured will be overall response rate, urodynamic tests, clinical assessment, and safety assessments. The methodological quality of this Bayesian-based network meta-analysis will be conducted with the “Risk of Bias” tool. Stata14.0 and WinBUGS 1.4.3 will be used to analyze the data. Furthermore, the assessment of heterogeneity, inconsistency, subgroup, sensitivity, and publication bias will also be taken into consideration with the help of Cochrane Collaboration's tool.

**Results:**

The findings of this study will be submitted to a peer-reviewed journal for publication.

**Conclusion:**

This work will furnish evidence-based recommendations to figure out the optimal Chinese medicine therapy or their combinations for NB induced by SCI, and in turn contribute to further research and public health.

## 1 Introduction

Neurogenic bladder (NB), characterized by voiding dysfunction of bladder and/or urethra, is a refractory disease caused by lesions affecting the nervous system.^[1]^ Spinal cord injury (SCI) is a common cause of NB. With the rapid development of urban construction, modern transportation, and sports practice, there has been an increasing incidence of SCI in recent years, particularly induced by trauma.^[2]^ According to statistics, there are 12 to more than 65 cases per million people suffer from SCI worldwide each year, mostly in young men and older adults. Meanwhile, around 70% to 84% of patients with SCI subject to NB.^[3]^ As the disease progresses, the damage and/or infection of upper urinary tract (e.g., renal failure) resulting from lower urinary tract dysfunction has become the leading cause of death in these patients.^[4]^ Therefore, NB after SCI has become a serious issue of concern. It not only has a severe impact on the quality of life of patients and their families, but also places a huge burden on the public health system.^[5]^

Management of NB after SCI has always been a major concern for practitioners. Conservative management, which includes intermittent catheterization (IC), timed voiding, micturition maneuvers, indwelling catheters, health education, and medications (e.g., anti-cholinergic medications), is still the most common treatment option, while surgical management can be used for those who are intolerant or failed to conservative treatment.^[6]^ The purpose of all treatments is to improve bladder function, in turn, protect the kidneys, and enhance the quality of life as well as societal value of the patients.^[7]^ However, IC, micturition maneuvers, and indwelling catheters require patients to have good self-care capabilities, otherwise infection is prone to occur. Besides, medications inevitably have some side effects and tolerability, while invasive surgery also has limitations such as high cost and high risk. Thus, it is still necessary to find other complementary and alternative treatments that are safe and effective.

Chinese medicine, which originated in China, has been widely practiced in East Asia for thousands of years. It is beyond doubt that Chinese medicine has made a great contribution to human health, especially in ancient times. However, as the times go by, the development of Chinese medicine has lagged behind. Nevertheless, Chinese medicine is gaining attention as a complementary and alternative medicine due to its good tolerability, few side effects, and low cost.^[8]^ In fact, the efficacy of Chinese medicine treatments for some refractory disease has been demonstrated.^[9–12]^ There are at least 18 interventions of Chinese medicine based on the guidelines developed by State Administration of Chinese medicine of China^[13]^. Many of these technologies have already been applied extensively in the treatment of NB after SCI, and some have shown promising results.^[14]^ However, owing to the variable severity of disease and the differences in quality of studies, the efficacy of different interventions varies. Thus, it makes sense to identify the optimal intervention or their combinations for NB after SCI.

In our study, we will conduct a Bayesian network meta-analysis to compare the efficacy and safety of different interventions of Chinese medicine in the treatment of NB after SCI. As far as we know, it will be the first attempt in this field. We believe this work will not only help patients recover better, but also provide practitioners with evidence-based evidence that will lead to greater savings in healthcare resources.

## 2 Methods

### 2.1 Study registration

Our study has been registered on the website of INPLASY, and acquired the registration number (INPLASY202180084). This network meta-analysis (NMA) will be conducted strictly under the guidance of PRISMA-P and PRISMA-NMA.^[15,16]^ Besides, the modifications of our NMA will be updated in detail timely.

### 2.2 Ethics

Considering that this NMA did not include the collection of personal information, ethical permission was not needed for this study.

### 2.3 Selection criteria

#### 2.3.1 Types of studies

All randomized controlled trials (RCTs) of testing Chinese medicine therapies for NB after SCI will be eligible for our study without any limitation of population characteristics. However, the language is limited to English or Chinese, and non-RCTs such as meeting abstracts, clinical experience, system reviews, case reports, and animal trials will be removed. Apart from this, sufficiency of original data is indispensable.

#### 2.3.2 Types of participants

The patients who are diagnosed with NB after SCI by a clear and widely recognized criterion will be recruited, and irrespective of gender, age, nationality, race.

#### 2.3.3 Types of interventions

##### 2.3.3.1 Experimental groups

Chinese medicine interventions or their combinations, based on the classification criteria of State Administration of Chinese medicine of China, must be applied in the experimental groups and regardless of differences in the specific prescription, duration, or frequency of treatments. In order to align with clinical practice and increase generalizability of our findings, we will merge similar therapies into 10 types of interventions (acupuncture therapy, electroacupuncture therapy, acupoint sticking therapy, acupoint injection therapy, moxibustion therapy, tuina/massage therapy, oral Chinese herb medicine, herb fumigation/soaking therapy, scrapping therapy, cupping therapy), which have been shown in detail in Table 1.

**Table 1:** The detailed explanation of postmerger Chinese medicine interventions.

Interventions	Explanation
Acupuncture therapy	Acupuncture is a manual technique in which fine metal needles are inserted into acupoints on the body to treat diseases.
Electroacupuncture therapy	Electroacupuncture is a kind of acupuncture therapy that combines conventional acupuncture with electrical stimulation.
Acupoint sticking therapy	The medications are made into certain dosage forms and applied to the acupoints.
Acupoint injection therapy	It involves injecting low-dose of medications into acupoints to treat diseases through the dual effects of drugs and acupoints.
Moxibustion therapy	Moxa is made into sticks or granules and then ignited. Subsequently, smoked it directly or indirectly on acupoints or certain areas of the body surfaces.
Tuina/massage therapy	It is a kind of Chinese medicine manipulation method which acts on meridians/acupoints or certain areas of the body by point method, pressing method, tapping method and pushing method.
Oral Chinese herb medicine	To decoct disease-related Chinese herbal medicine prescriptions and taken orally
Herb fumigation/soaking therapy	Fumigation or soaking of the affected area with warm Chinese herbal medicine liquids
Scrapping therapy	Blunt-edged instruments, such as horns, stone spoons, or ladles, are used, then dipped them in mediums such as scraping oil, water, or lubricant, and repeatedly scraped on certain areas of the body surfaces to cause localized petechiae.
Cupping therapy	Cup is used as a tool, and by means of burning, suction, and steam, negative pressure is induced inside the cup, in turn, causing the cup to adsorb on acupoints or certain areas of the body surfaces, then local skin congestion or blood stasis will occur.

##### 2.3.3.2 Control groups

The control groups with non-Chinese medicine interventions, consisting of non- surgical conservative therapies (IC, indwelling catheters, pelvic-floor electro-stimulation, bladder training, medications, and so on) and blank control methods (placebo, sham acupuncture, and so on), will be included in our NMA.

#### 2.3.4 Types of outcomes

The primary outcome of our NMA will be measured by overall response rate and urodynamic tests, which includes postvoiding residual urine volume, maximum urinary flow rate, and maximal detrusor pressure. The secondary outcome includes a voiding diary (the number of patients with retention or incontinence, their average number of urination or incontinence episodes per 24 hours), clinical assessments (e.g., 1 h/24 h pad test), quality of life questionnaire. Apart from this, safety assessments, for instance, drop-out cases and adverse events will be considered as well.

### 2.4 Database and search strategy

A comprehensive search for potentially relevant literature will be performed in 8 online databases from their establishment throughout June 2021: the Cochrane Library, Web of Science, PubMed, EMBASE Database, China Biological Medicine Database, Chinese Scientific Journals Database, Wan Fang databases, and China National Knowledge Infrastructure. Search strategy based on MeSH terms combining with free text words will be applied in English databases, while counterpart terms in Chinese will be used in Chinese databases. Additionally, the related references of included literature will also be screened carefully under the guidance of the snowball strategy, and the language will be limited to English or Chinese. If the information we need is incomplete, we will contact the corresponding author to obtain it, and an intention-to-treat analysis will also be conducted for missing or unreachable data. The detailed retrieval strategy of PubMed is listed in Table 2 as an example.

**Table 2:** PubMed search strategy draft.

Number	Search items
#1	“Urinary Bladder, Neurogenic”[Mesh]
#2	Bladder, Neurogenic[Title/Abstract] OR Neurogenic Bladder[Title/Abstract] OR Urinary Bladder Neurogenic Dysfunction[Title/Abstract] OR Neurogenic Dysfunction of the Urinary Bladder[Title/Abstract] OR Neurogenic Urinary Bladder Disorder[Title/Abstract] OR Neuropathic Bladder[Title/Abstract] OR Urinary Bladder Disorder, Neurogenic[Title/Abstract] OR Neurogenic Bladder Disorder[Title/Abstract] OR Urinary Bladder Neurogenesis[Title/Abstract] OR Neurogenesis, Urinary Bladder[Title/Abstract] OR Bladder Neurogenesis[Title/Abstract] OR Neurogenesis, Bladder[Title/Abstract] OR Neurogenic Urinary Bladder, Atonic[Title/Abstract] OR Neurogenic Bladder, Atonic[Title/Abstract] OR Atonic Neurogenic Bladder[Title/Abstract] OR Neurogenic Urinary Bladder, Spastic[Title/Abstract] OR Neurogenic Urinary Bladder, Uninhibited[Title/Abstract]
#3	#1 OR #2
#4	“Spinal Cord Injuries”[Mesh]
#5	Spinal Cord Trauma[Title/Abstract] OR Myelopathy, Traumatic[Title/Abstract] OR Traumatic Myelopathies[Title/Abstract] OR Spinal Cord Transection[Title/Abstract] OR Spinal Cord Laceration[Title/Abstract] OR Post-Traumatic Myelopathy[Title/Abstract] OR Post Traumatic Myelopathy[Title/Abstract] OR Spinal Cord Contusion[Title/Abstract]
#6	#4 OR #5
#7	“Medicine, Chinese Traditional”[Mesh]
#8	“Acupuncture”[Mesh]
#9	“Electroacupuncture”[Mesh]
#10	“Acupuncture Points”[Mesh]
#11	“Moxibustion”[Mesh]
#12	“Massage”[Mesh]
#13	“Drugs, Chinese Herbal”[Mesh]
#14	“Cupping Therapy”[Mesh]
#15	Zhong Yi Xue[Title/Abstract] OR Medicine, East Asian Traditional[Title/Abstract] OR Acupuncture Therapy[Title/Abstract] OR Acupoint[Title/Abstract] OR trigger Points[Title/Abstract] OR Moxabustion[Title/Abstract] OR Tui na[Title/Abstract] OR Chinese Drugs, Plant[Title/Abstract] OR Herbal Drugs, Chinese[Title/Abstract] OR Plant Extracts, Chinese[Title/Abstract] OR Cupping Treatment[Title/Abstract] OR Acupoint sticking[Title/Abstract] OR Acupoint injection[Title/Abstract] OR Herb fumigation[Title/Abstract] OR Herb soaking[Title/Abstract] OR Scrapping[Title/Abstract]
#16	#7 OR #8 OR #9 OR #10 OR #11 OR #12 OR #13 OR #14 OR #15
#17	“Randomized Controlled Trial”[Publication Type]
#18	“Randomized Controlled Trials as Topic”[Mesh]
#19	“Pragmatic Clinical Trial”[Publication Type]
#20	“Pragmatic Clinical Trials as Topic”[Mesh]
#21	“Intention to Treat Analysis”[Mesh]
#22	“random allocation”[Mesh Terms]
#23	random^∗^[Title/Abstract]
#24	#17 OR #18 OR #19 OR #20 OR #21 OR #22 OR #23
#25	#3 AND #6 AND #16 AND #24

### 2.5 Literature selection and data extraction

**Figure 1:**
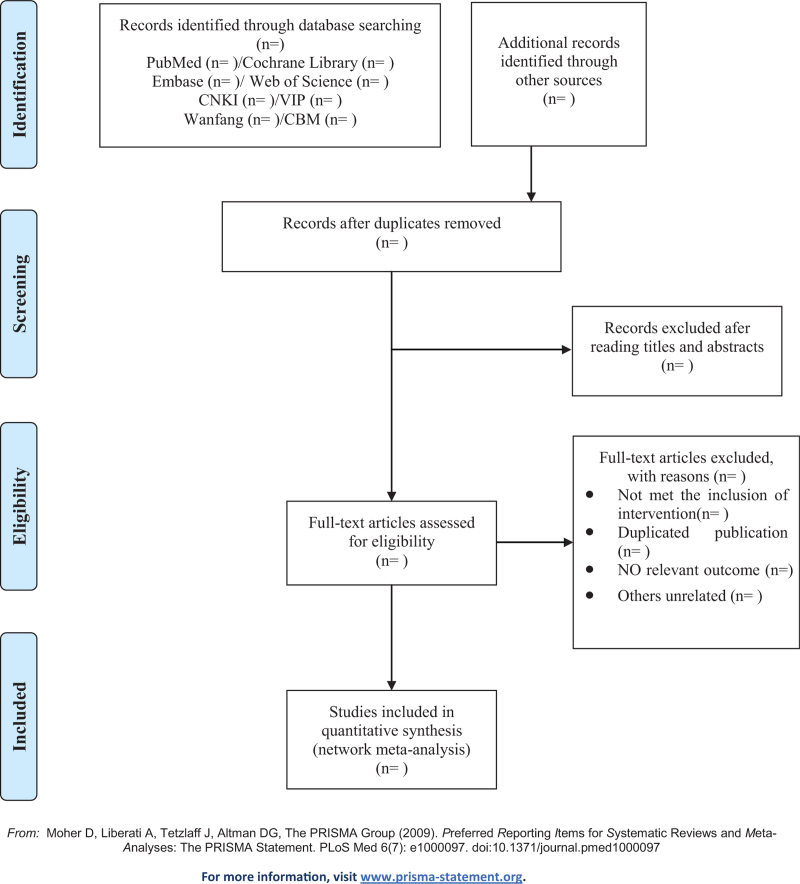
Flow chart of study selection.

The first step is to import the retrieved literature into EndNote X8 to remove duplicates automatically, then primary screening will be performed independently by 2 reviewers (Zhihong Zhu and Haitao Jin) based on titles and abstracts, and studies not meeting the selection criteria will be removed directly. The second step is to conduct a full-text screening to select eligible articles by the same 2 reviewers, and the exclusion reasons will be recorded individually. The third step is to carry out a cross-checking of the results to ensure the consistency of the screening. When the disagreements appeared, a third senior assessor (Yue Zhuo) will be asked to assist in the ultimate judgement. The specific selection process is illustrated in Figure 1.

When the above steps are completed, a data extraction table, which mainly includes first author's name, nationality, publication year, participants’ characteristics (sample size, gender, mean age, number of groups, type of NB after SCI, disease duration, and so on), interventions, comparators, outcomes, and methodological design will be established in Microsoft Excel 2016 in light of recommendations in the Cochrane Handbook. Given that baseline situation among different trials may be potentially varying, the data finally included in the analysis were estimated by the following formula, which was recommended in the Cochrane Handbook for Systematic Reviews of Interventions (version5.1) and r is a correlation coefficient with a value of 0.5.^[17]^ This approach allowed for a better presentation of the changes before and after treatment of dissimilar Chinese medicine interventions.

(1) $${\bar{x}}_{change}={\bar{x}}_{post-treatment}-{\bar{x}}_{baseline}$$

(2) $$SD_{change}=\sqrt{{\left(SD_{baseline}\right)}^{2}+{\left(SD_{post-treatment}\right)}^{2}-2\times r\times SD_{baseline}\times SD_{post-treatment}}$$

### 2.6 Quality assessment

Two well-trained researchers (Boyu Wu and Yue Zhuo) will independently assess the bias risk of all the included RCTs by the Cochrane Collaboration tool,^[18]^ which consisted of the following aspects: assignment concealment, random sequence generation, blinding of outcome assessors, blinding of participants and personnel, selective reporting, the integrity of outcome data, and other sources of bias. Each field has been classified as high risk, low risk, or unclear risk. Any disagreements will be resolved by discussion with a third senior assessor (Zhijie Li).

### 2.7 Statistical analysis

First of all, a pairwise meta-analysis will be performed using Revman 5.3. (Cochrane Collaboration, Oxford, UK) for the direct comparisons. Secondly, considering the anticipated heterogeneity, the NMA within a Bayesian framework will be conducted by WinBUGS 1.4.3 (MRC Biostatistics Unit, Cambridge, UK) based on the random effect model for the results of the indirect comparison. Besides, models will be calculated with Markov chain Monte Carlo algorithm^[19]^: 4 chains will be employed for simulation analysis, the step size will be set to 10, the number of annealing times will be set to 20,000 for reducing the impact on arbitrary values, and the number of iterations will be set to 50,000. Additionally, the continuous outcomes will be measured by standard mean difference with 95% confidence interval for indirect comparisons, while binary variable selection relative risk and 95% confidence interval. Thirdly, the plot of surface under the cumulative ranking curve will be computed by STATA 14.0. (Stata Corporation, College Station, Texas) to forecast the possible ranking order.^[20]^ In our study, a higher surface under the cumulative ranking curve score represents the better Chinese medicine intervention for NB after SCI.^[21]^

### 2.8 Assessment of heterogeneity

First of all, we will assess if the participants’ characteristics, interventions, as well as outcomes are adequately similar in all included trials. If not, the heterogeneity of the results will be assessed with I-square (I^2^) by STATA 14.0. software. The substantial heterogeneity will be considered only if the I^2^ is above 50%, and then a random-effects model will be chosen, otherwise a fixed-effects model will be used.

### 2.9 Assessment of inconsistency, subgroup, and sensitivity

In general, differences across dissimilar kinds of evidence may be a major source of inconsistency. Thus, the inconsistency between indirect and direct evidence will be assessed by the loop inconsistency test and node-splitting method. Additionally, Z-value as well as corresponding *P* value will be calculated, and *P* values less than .05 demonstrate a significant difference.

In case significant heterogeneity is identified, subgroup analysis will be performed according to the possible sources of heterogeneity, such as type of NB after SCI (retention or incontinence), injured spinal segments, and quality of studies.

Considering that varying level of the methodological quality of trails may tend to affect our findings, sensitivity analysis will be performed to evaluate the robustness of the results by excluding high-risk studies.

### 2.10 Assessment of publication bias

In our NMA, we will strictly adhere to the mentioned rules of literature selection to minimize the potential impact of publication bias. Then, if the number of included trails is above 10, a funnel plot will be employed to assess the publication bias visually.

### 2.11 Grading the quality of evidence

Two reviewers will evaluate the quality of each evidence separately on the basis of the GRADE working group methodology,^[22]^ which classifies the quality levels into 4 categories: high, medium, low, and very low. Similarly, a third one will be consulted, if there is uncertainty.

## 3 Discussion

Urine storage and voiding requires the precise regulation of the central and peripheral nervous system, and different injured spinal segments may lead to dissimilar types of micturition dysfunction.^[23]^ It has been reported that urologic disease after SCI, NB in particular, is the complication that most affects patient's quality of life.^[24]^ Chinese medicine has the advantages of diverse methods, extensive experience, and easy operation. As a result, increasing attention has been paid to the clinical effects and potential mechanisms of Chinese medicine for NB.^[14,25]^ For instance, electroacupuncture has been reported to exhibit an important role in the improvement of micturition reflex after SCI, and in turn accelerating the recovery of the bladder function.^[26]^ Tanshinone IIA, the active ingredients of Chinese herb “Danshen” (*Salvia miltiorrhiza Bunge*), has been found to improve urodynamic parameters and bladder functional recovery after SCI induced NB by reducing the inflammation and edema of the bladder tissue as well as enhancing spinal cord regeneration.^[27]^ However, gaps in comparisons of direct and indirect evidence of various Chinese medicine therapies still remain, which confuse clinical practice. Trying to address this issue by NMA is our original intention.

Predictably, some potential limitations may affect the credibility of our study. Due to a restriction of languages, selection bias is inevitable, which may limit the generalization of our conclusions. Furthermore, to some extent, it is hard for us to rule out heterogeneity completely because of the differences in specific prescription (e.g., acupoint selections, medical manipulations) and treatment frequency. Nevertheless, this NMA will be conducted on the basis of adequate high-quality trials, and strictly in line with Cochrane Manual. We are looking forward to provide the clinical and experimental research of NB after SCI with evidence-based recommendations.

## Author contributions

**Conceptualization:** Zhihong Zhu, Zhijie Li.

**Data curation:** Zhihong Zhu, Haitao Jin.

**Formal analysis:** Zhihong Zhu, Yue Zhuo, Boyu Wu.

**Methodology:** Yue Zhuo, Boyu Wu.

**Supervision:** Zhijie Li.

**Writing – original draft:** Zhihong Zhu.

**Writing – review & editing:** Zhihong Zhu.
